# Production of Monoclonal Antibodies against Human Trefoil Factor 3 and Development of a Modified-Sandwich ELISA for Detection of Trefoil Factor 3 Homodimer in Saliva

**DOI:** 10.1186/s12575-017-0064-3

**Published:** 2017-11-08

**Authors:** Saichit Khummuang, Waraporn Phanphrom, Witida Laopajon, Watchara Kasinrerk, Ponlatham Chaiyarit, Supansa Pata

**Affiliations:** 1 Division of Clinical Immunology, Department of Medical Technology, Faculty of Associated Medical Sciences, Chiang Mai University, Chiang Mai, 50200 Thailand; 2 Biomedical Technology Research Center, National Center for Genetic Engineering and Biotechnology, National Science and Technology Development Agency at the Faculty of Associated Medical Sciences, Chiang Mai University, Chiang Mai, 50200 Thailand; 3 Department of Oral Diagnosis, Faculty of Dentistry, Khon Kaen University, Khon Kaen, 40002 Thailand; 4 Research Group of Chronic Inflammatory Oral Diseases and Systemic Diseases Associated with Oral Health, Khon Kaen University, Khon Kaen, 40002 Thailand

**Keywords:** Monoclonal Antibody, Modified-sandwich ELISA, Trefoil Factor, Saliva, Oral Diseases, Homodimeric Peptides

## Abstract

**Background:**

Human trefoil factor (TFF) peptides consist of three members: TFF1, TFF2 and TFF3. TFF3 is the most abundant TFF peptide in saliva. TFF3 homodimer was suggested to be involved in apoptosis inhibition and malignancy. Determination of TFF3 homodimer expression profiles in saliva may lead to new information about oral biology and diseases. The objective of this study was to generate monoclonal antibodies (mAbs) against TFF3 and apply the produced mAbs for the establishment of ELISA for quantification of dimeric TFF3 in saliva.

**Results:**

With our modified hybridoma technique, three hybridoma clones producing anti-TFF3 mAbs having IgG isotype were generated. The mAbs were specific for TFF3 with no cross-reactivity to other TFFs. Using the generated mAbs, a modified-sandwich ELISA with high sensitivity for the quantification of dimeric TFF3 in saliva was developed. Using this ELISA, the amount of dimeric TFF3 in saliva could be measured.

**Conclusions:**

A modified-sandwich ELISA for the quantification of TFF3 dimeric form was established. The established ELISA will be a valuable tool for facilitating the investigation of the physiological roles and the diagnostic values of TFF3 in oral diseases. The concept of this modified-sandwich ELISA may be applied for the determination of other homodimeric peptides of interest.

## Background

Trefoil factor (TFF) is a small soluble peptide containing a three-leaved structure called the TFF domain. Human TFF peptides consist of three members: TFF1, TFF2 and TFF3, with molecular weights of 6.5 kDa, 12 kDa and 6.6 kDa, respectively [1–3]. TFF peptides are secreted by mucin-producing epithelial cells [4–7]. Various functions of TFFs have been demonstrated including wound repair, cell migration, cell proliferation, anti-apoptosis, regeneration, neovascularization, mucin interaction and immunomodulation [8–10].

Human TFF3 is mainly secreted by intestinal epithelial and goblet cells. The TFF3 is also expressed by the uterus, breast, some parts of the hypothalamus, pituitary glands in the brain and oral tissue [11–16]. The human TFF3 contains a trefoil domain and is capable of forming dimerization via a disulphide bond involving the cysteine residue located near the C-terminus. Upon dimerization, the additional 3(10)-helix involving residues 53–55 occur in the TFF3 homodimer which contribute to the distinct structure of homodimer and monomer [17]. From functional viewpoints, TFF3 monomer induces cell migration by activating the mitogen-activated protein kinase (MAPK) pathway independently of the epidermal growth factor receptor (EGFR) [18–20]. In contrast, depending on EGFR and nuclear factor-kappa B (NF-κB) signaling pathway, TFF3 homodimer has an anti-apoptotic effect on epithelial cells [20–23]. Furthermore, it has been demonstrated that TFF3 which interacts with other peptides also has distinct functions [24, 25].

Regarding oral compartments, TFF peptides have been detected in salivary glands [11, 12, 14, 16], gingiva [26], oral mucosa [27–29] and saliva [26, 27, 30, 31]. Our previous studies have demonstrated that TFF3 is the most abundant TFF peptide in saliva, followed by TFF1 and TFF2 [26, 30]. TFF3 peptides have been reported to be involved in several oral biological functions [10, 32]. It is a modifying factor for signaling pathways in cell survival, cell proliferation and cell migration of oral keratinocytes [33, 34]. TFF3 expression has been found to be significantly decreased in chronic periodontitis [26], oral cancer [28] and oral lichen planus [29], but significantly increased in benign and malignant salivary gland tumors [16]. However, an explanation for the changes in the expression of TFF3 in various oral diseases remains unclear. Research regarding TFF3 is, therefore, an emerging feature in the dental field [10]. As disulfide-linked homodimeric TFF3 is involved in anti-apoptosis, the study of TFF3 homodimer expression profiles in oral secretions such as saliva and gingival crevicular fluid may bring new insights into oral biology and diseases. At present, several techniques for the quantitative measurement of TFF3 have been developed, including gene expression, immunohistochemistry, mass spectrometry and Western blotting [35–38]. Unfortunately, all developed methods are time-consuming, require highly skilled personnel and are not practical for use in mass screening. Although ELISA for TFF3 detection and measurement has been developed, the technique cannot distinguish between dimeric and monomeric TFF3.

In this study, we sought to develop an immunoassay for the determination of TFF3 homodimer in saliva. We have produced mAbs specific for human TFF3 peptides using our modified hybridoma technique [39]. Using the generated mAb, a modified-sandwich ELISA was developed which could be employed for the detection of TFF3 homodimer in human saliva.

## Results

### Production and Characterization of Monoclonal Antibodies against TFF3 Peptide

**Fig. 1:**
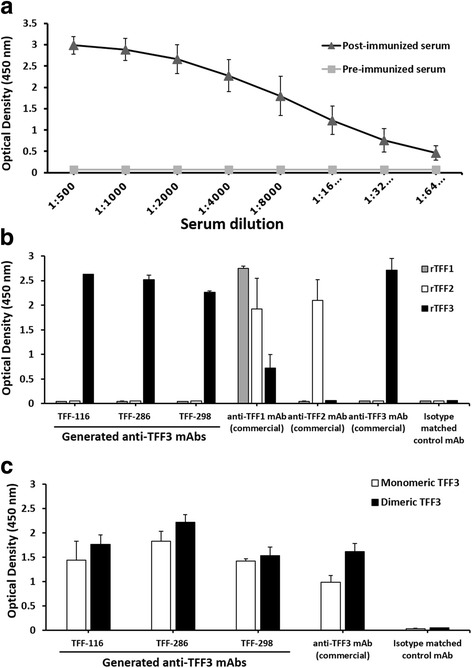
Antibody responses of immunized mouse and characterization of anti-TFF3 monoclonal antibodies. **a** Mouse was immunized with recombinant TFF3 three times at two-week intervals. Blood was collected before (pre-immunization: square marker and gray line) and 7 days after the third immunization (post-immunization: triangle marker and black line). Various dilutions (as indicated) of the mouse sera were investigated for the presence of anti-TFF3 antibodies by indirect ELISA. Values are presented as mean ± SD of two independent experiments. Paired, two tailed Student’s *t* test was used to assess significance level of anti-TFF3 antibody in pre-and post-immunization sera (*P* < 0.01). **b** Indirect ELISA was performed for the determination of the specificity of the generated anti-TFF3 mAbs (TFF-116, TFF-286 and TFF-298). Human recombinant (r) TFF1, TFF2 and TFF3 or (**c**) human recombinant monomeric and dimeric TFF3 were coated on an ELISA plate. Commercial anti-TFF1, anti-TFF2 and anti-TFF3 mAbs were used as positive controls for reacting with their corresponding antigens. IgG1,κ was used as the isotype-matched control mAb (isotype control). Bar graphs represent mean ± SD of two independent experiments. There was statistically significantly higher reactivity in tested mAb compared with isotype matched control mAb (all *P*-values <0.05)

To establish the hybridoma producing mAb against the human TFF3 peptide, a mouse was immunized with recombinant dimeric TFF3. High anti-TFF3 antibody response was detected after antigen immunization (Fig. 1a). The mouse spleen cells were then collected. The IgG expressing cells were isolated from the spleen cells and used for generation of hybridoma cells by hybridoma technique. Three anti-TFF3 antibody-producing hybridoma clones named TFF-116, TFF-286 and TFF-298 were generated. All of the generated mAbs were IgG1/κ isotypes.

**Fig. 2:**
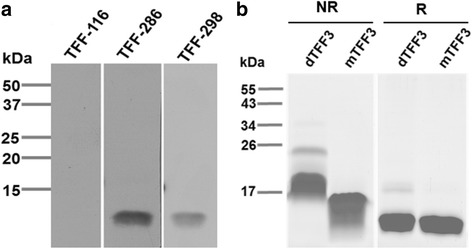
Western blot analysis of anti-human TFF3 monoclonal antibodies. **a** The Western blotting results were demonstrated with the indicated anti-TFF3 mAbs using human recombinant monomeric TFF3 under reducing conditions. **b** SDS-PAGE demonstrated the molecular size of human recombinant monomeric TFF3 (mTFF3) and dimeric TFF3 (dTFF3) in non-reducing (NR) and reducing (R) conditions. The proteins were stained using PageBlue™ Protein Staining Solution. The molecular markers (kDa) are indicated on the left. The data was representative of two independent experiments

The mAbs reacted strongly to the recombinant TFF3 without cross-reactivity to the recombinant TFF1 and TFF2 peptides (Fig. 1b). The produced anti-TFF3 mAbs were then tested for their reactivity to the monomeric and the dimeric forms of TFF3. It was observed that all the anti-TFF3 mAbs reacted to both forms of the TFF3 peptide in the same manner as the commercial anti-TFF3 mAb (Fig. 1c). The Western blotting experiments were carried out under reducing conditions. Two mAbs (TFF-286 and TFF-298) showed positive reactivity with a protein band (Fig. 2a) at the same molecular size of reduced recombinant monomeric TFF3 observed in SDS-PAGE (Fig. 2b), which is in accordance with a previous report [38]. However, it was observed that mAb TFF-116 produced no visible band (Fig. 2a), which indicates that mAb TFF-116 might react to the conformational epitope of the TFF3 peptide.

### Modified-Sandwich ELISA for Detection of TFF3 Homodimer

**Fig. 3:**
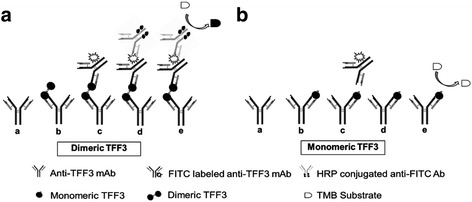
Schematic diagram demonstrating the principle behind the developed sandwich ELISA detecting dimeric TFF3. **a** The steps of the developed sandwich ELISA: (**a**). First, anti-TFF3 mAb is coated on the ELISA plate. **b**. Human recombinant TFF3 or saliva is added. The TFF3 present in the sample binds to the mAbs. **c**. The FITC-conjugated secondary anti-TFF3 mAb (which is the same clone of the coated mAb) is added. d. HRP-conjugated anti-FITC antibody is added and there is a reaction to the FITC-labeled anti-TFF3 mAb. e. Lastly, the TMB substrate is added and the color develops. The intensity of the color is proportional to the amount of the dimeric TFF3 present in the sample. Dimeric TFF3 can bind to both the coated mAb and the secondary mAb and the colorimetric signal can be developed by the reaction of the HRP-conjugated anti-FITC antibody with the TMB substrate. **b** In contrast, the secondary mAb cannot bind to monomeric TFF3 which cannot develop the colorimetric signal

In order to develop an effective ELISA for the quantification of the TFF3 dimeric form, a sandwich-type ELISA was chosen to be the assay system. As the TFF3 monomer is a small peptide, we decided to use the same mAb both for capturing the TFF3 and for tracking the captured TFF3 in the developed sandwich ELISA. This, therefore, will ignore the detection of the monomeric form and detect only the dimeric form. In order to enhance the sensitivity, FITC and anti-FITC detection systems were employed in the sandwich ELISA. An anti-TFF3 mAb was used as the first antibody for coating plates in order to capture the TFF3 peptide in the samples. The same anti-TFF3 mAb labeled with FITC was used to detect the bound TFF3. The HRP-anti-FITC conjugates were added to the system to detect the binding of the FITC-labeled antibodies on the plate. A graphic representation of the developed sandwich ELISA for TFF3 homodimer quantification is shown in Fig. 3.

**Fig. 4:**
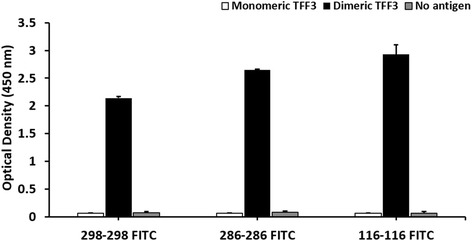
Sandwich ELISA for the measurement of TFF3 homodimer**.** The sandwich ELISA was developed using a pair of mAbs against TFF3. The three combinations of the capture and detector mAbs, namely TFF-116 and FITC-labeled TFF-116 (116–116 FITC), TFF-286 and FITC-labeled TFF-286 (286–286 FITC) and TFF-298 and FITC-labeled TFF-298 (298–298 FITC), were employed for detecting recombinant TFF3 monomeric and dimeric forms. Bar graphs represent mean ± SD of two independent experiments. There was statistically significantly higher reactivity in dimeric form compared with monomeric and no antigen (all *P*-values <0.05)

In our experiments, three different pairs of anti-TFF3 mAbs (clones TFF-116, TFF-286 and TFF-298) were studied in order to obtain the optimal condition for the measurement of the TFF3 dimeric form. Using mAb clones TFF-116, TFF-286 and TFF-298, the developed sandwich ELISA could determine the TFF3 dimeric, but not the monomeric form (Fig. 4).

**Fig. 5:**
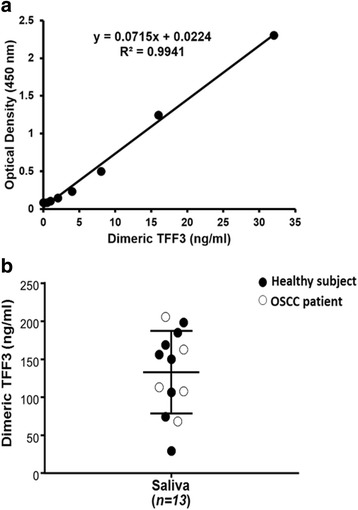
Detection of human TFF3 homodimer in saliva. **a** Various concentrations of the dimeric TFF3 were measured by the developed sandwich ELISA using TFF-286 as the capture and detector mAbs. A typical calibration curve covering TFF3 concentrations in the range of 0.5–32 ng/ml was obtained. **a** representative result from one of five experiment is shown. **b** Salivary TFF3 concentrations were determined by the developed sandwich ELISA. The levels of the salivary TFF3 concentrations in saliva (*n* = 13) are shown. Eight healthy subjects (closed circle) and five oral squamous cell carcinoma (OSCC) patients (open circle) were indicated. The data was representative of two independent experiments

The hybridoma clone TFF-286, which produces anti-TFF3 mAb more actively than other clones, was selected and employed in the developed ELISA. Using mAb TFF-286 as the capture and FITC-labeled mAb, a standard curve detecting TFF3 in the range of 0.5 ng/ml to 32 ng/ml was obtained (Fig. 5a). Within five independent experiments, the linear regressions of standard curves ranged from 0.954 to 0.997. The developed ELISA was employed to determine TFF3 in saliva samples of both healthy subjects and oral squamous cell carcinoma (OSCC) patients. The levels of TFF3 of the tested subjects could be determined at 134 ± 58.44 ng/ml (mean ± SD) (Fig. 5b).

## Discussion

TFF3 is predominantly secreted by intestinal epithelial [40] and goblet cells [41]. Interestingly, along with being a member of the TFF family, TFF3 was found to be abundantly expressed in human saliva [26, 27, 30]. Previous studies have demonstrated the altered expression of TFF3 in saliva and in oral tissues from patients with various oral diseases [16, 26, 28, 42]. Investigation of TFF3 is, therefore, an emerging aspect in the field of oral biology. In this study, we intended to develop a novel immunoassay for the determination of TFF3 homodimer in saliva.

The IgG mAbs usually have higher affinity compared to the IgM isotype and are more advantageous for applications in immunological methods [43]. In order to produce mAbs with the desired isotypes, we modified the standard hybridoma technique for the production of mAbs having a desired isotype [39]. In the present study, we aimed to generate IgG mAbs specific for the TFF3 peptide. The modified hybridoma technique was then employed. Using the modified hybridoma technique, the cells expressing the surface IgG were first isolated from the spleen cells of the immunized mouse and subjected to cell fusion in order to produce hybridoma clones. As intended, three anti-TFF3 mAbs could be generated, all of which were the IgG1 isotype. The produced mAbs showed high specificity to TFF3 without cross-reactivity to TFF1 and TFF2.

A number of studies have demonstrated that TFF3 dimeric form is anti-apoptotic which is very important for epithelial restitution [10, 17, 20–23]. The most significant structural differences between the trefoil domains in the TFF3 monomer and dimer are in the additional 3(10)-helix (residues 53–55) outside of the core region [17]. A slight difference in the monomeric and the dimeric forms of TFF3 was predicted. The TFF3 monomeric and dimeric forms were directly coated on the ELISA plate by indirect ELISA to confirm whether the produced mAbs reacted to the TFF3 monomer or the TFF3 dimer. All the generated anti-TFF3 mAbs reacted to the dimeric as well as the monomeric forms of TFF3. The results indicate that the produced mAbs react to epitopes which are presented in the TFF3 peptide. These epitopes are not altered upon TFF3 dimerization. None of the produced anti-TFF3 mAbs, therefore, could distinguish between the monomeric and dimeric forms.

Currently, several techniques for the quantitative measurement of TFF3 have been developed. Among available TFF3 detection methods, ELISA is the most suitable method for the detection of secreted TFF3, especially in saliva. Various ELISA kits for detecting TFF3 are available. An ELISA which was developed using polyclonal antibodies against TFF3 has been claimed to determine the dimeric form of TFF3 [44], but its specificity is doubtful. In addition, for polyclonal antibody production, the variation between the different batches of preparation is a concern [45]. The drawbacks of the available ELISA led us to consider the establishment of a more effective method for specifically determining TFF3 dimeric form. In this study, a sandwich ELISA method for the measurement of the TFF3 homodimer was developed using the produced anti-TFF3 mAb. In developing our sandwich ELISA procedure, the anti-TFF3 mAb was coated on the ELISA plate to capture the TFF3 in the sample and the same anti-TFF3 mAb was used as a tracker to determine the captured TFF3. Instead of labeling the second mAb with an enzyme, we labeled it with a fluorescent dye, FITC. The FITC-anti-TFF3 mAb was then detected using the HRP-conjugated anti-FITC antibody. Essentially, our sandwich ELISA could detect only the TFF3 homodimer, not its monomeric form. The TFF3 monomer is a short peptide, in which the binding of a capture mAb completely occupies the epitope on the single trefoil domain, resulting in the blockage of the second specific mAb binding. In contrast, the dimeric form contains a double trefoil domain and has more epitopes available for the second mAb binding. This phenomenon, therefore, indicates that our sandwich ELISA was able to determine only the dimeric form of the TFF3 in the sample. Using this system, a standard curve for quantifying TFF3 could be established with very high sensitivity. This is required because there is no information about the natural form of TFF3 in saliva. The developed sandwich ELISA was then employed to determine the dimeric TFF3 in saliva. The dimeric form of TFF3 could be detected in saliva samples by the established modified-sandwich ELISA.

## Conclusions

In this study, we have employed a modified hybridoma technique to produce anti-TFF3 mAbs having IgG isotype. Using the produced mAb, a sandwich type ELISA was developed for the detection of homodimeric TFF3 in saliva. The developed ELISA will be a valuable tool for the investigation of the physiological and pathological roles of TFF3 in health and diseases. It may also have diagnostic value for oral diseases. In addition, the concept of the presented ELISA platform may be applied for the measurement of other homodimer peptides.

## Methods

### Hybridoma Production

A BALB/C mouse was intraperitoneally (IP) immunized with human recombinant dimeric TFF3 (kindly provided by Dr. Lars Thim, Novo Nordisk A/S, DK-2880 Bagsvaerd, Denmark) [46] three times at two-week intervals. Sera were collected and tested for the presence of anti-TFF3 antibody by indirect ELISA. After a high level of antibody titer was detected, the mouse was IP boosted with 50 μg dimeric TFF3 in the absence of Freund’s adjuvant. Five days after the last boosting, the mouse was sacrificed and its spleen cells were collected and fused with myeloma cells by modified hybridoma technique [39]. In brief, the spleen cells were subjected to isolate cells expressing surface IgG using a Magnetic Cell Sorting System (MACS) (Miltenyi Biotec, Bergish Gladbach, Germany). The isolated cells were fused with myeloma cells (P3-X63Ag8.653) by a standard hybridoma technique using 50% polyethylene glycol (Sigma-Aldrich). After the HAT medium (Sigma-Aldrich) selection, the culture supernatants obtained from the hybridoma-containing wells were analyzed for antibody reactivity by indirect ELISA. Single cell cloning of the positive wells was performed by the limiting dilution technique. The isotype of the produced mAbs was determined using an IsoStrip mouse monoclonal antibody isotyping kit (Roche, Penzberg, Germany). In brief, culture supernatants obtained from the cloned hybridomas were added into the tube containing blue-latex beads bearing anti-mouse kappa and anti-mouse lambda antibodies. The reactions were then inserted to immunochromatographic strip test which contained an immobilized band of goat anti-mouse antibodies regarding to common mouse antibody isotypes (IgG_1_, IgG_2a_, IgG_2b_, IgG_3_, IgA and IgM) and kappa and lambda light chains. The blue-latex beads aggregated as the blue band, corresponding to the immunoglobulin isotype and light chain of mAb.

### Purification of Monoclonal Antibodies

Hybridomas producing anti-TFF3 mAbs were cultured in serum-free condition using a hybridoma serum-free medium (Gibco, Grand Island, NY, USA). The mAbs were purified from culture supernatants by affinity chromatography using HiTrap Protein G columns (GE Healthcare Bio-Sciences AB, Uppsala, Sweden). The obtained purified mAbs were checked for their purity and activity by sodium dodecyl sulfate polyacrylamide gel electrophoresis (SDS-PAGE) and indirect ELISA, respectively. The purified mAbs were kept at −20 °C.

### SDS-PAGE and Western Blotting

Recombinant monomeric and dimeric TFF3, kindly provided by Dr. Lars Thim [46], were separated in non-reducing (62.5 mM Tris-HCl pH 6.8, 2.5% SDS, 0.002% bromphenol blue, 10% glycerol) and reducing conditions (62.5 mM Tris-HCl pH 6.8, 2.5% SDS, 0.002% bromphenol blue, 5% β-mercaptoethanol (2-ME), 10% glycerol) and made to undergo SDS-PAGE using 15% polyacrylamide gel. The separated proteins were electrotransferred onto a nitrocellulose membrane (Millipore, Darmstadt, Germany). The membrane was blocked with 5% skimmed milk in phosphate buffer saline (PBS) at 4 °C overnight. The membranes were incubated with anti-human TFF3 mAbs for 1 h and washed with 0.05% Tween 20 in PBS. The membrane was then incubated with horseradish peroxidase (HRP)-conjugated rabbit anti-mouse immunoglobulin antibodies (DAKO, Glostrup, Denmark) for 1 h. The protein bands were developed by incubation with SuperSignal ECL substrate (Thermo Scientific) and visualized by exposure to X-ray films.

### Indirect ELISA

The recombinant TFF1, TFF2 and TFF3 were purchased from RayBiotech (Norcross, GA, USA). Recombinant monomeric TFF3 and dimeric TFF3 were kindly provided by Dr. Lars Thim [46]. The recombinant TFFs (1 μg/ml), monomeric (10 μg/ml) or dimeric TFF3 (10 μg/ml), were coated on an ELISA plate (Costar, Corning, NY, USA) using a carbonate/bicarbonate coating buffer pH 9.6 at 4 °C overnight. The plates were blocked with 2% skimmed milk in PBS at 37 °C for 1 h. Mouse sera, hybridoma culture supernatants, or commercial antibodies, including anti-TFF1 mAb (Sigma-Aldrich), anti-TFF2 mAb (R&D Systems, Minnesota, USA) and anti-TFF3 mAb (R&D Systems), were added and incubated at 37 °C for 1 h. The plates were washed and HRP-conjugated rabbit anti-mouse immunoglobulin antibodies (DAKO) were added; thereafter, the plates were incubated at 37 °C for 1 h. Subsequently, 3, 3′, 5, 5′-tetramethylbenzidine (TMB) substrate (Invitrogen, Camarillo, CA, USA) was added. The reaction was stopped using 1 N HCl and the absorbance was measured at OD450 nm.

### Saliva Preparation

Saliva samples from healthy donors and oral squamous cell carcinoma patients were collected in a tube and centrifuged for 5 min at 10,000 g. The aqueous layer was collected. The samples were assayed immediately or stored at −20 °C; repetition of freeze-thaw cycles was avoided.

### Labeling of Fluorescence Isothiocyanate (FITC) to Anti-TFF3 Monoclonal Antibodies

FITC (Sigma-Aldrich) was dissolved in anhydrous dimethylsulfoxide (DMSO) (Sigma-Aldrich) at 10 mg/ml. The purified anti-TFF3 mAbs (0.5–2 mg/ml) were subjected to pH adjustment by adding 1 M sodium bicarbonate (NaHCO_3_) of pH 9.4 to obtain the final concentration of 0.1 M NaHCO_3_. A volume of FITC solution was added to the purified mAbs. The volume of FITC was calculated as follows:

Volume of FITC (ml) = [(Total concentration of antibodies (mg) × 0.1) / Molecular weight of IgG (146,000)] × [Molecular weight of FITC (389.39) × constant value for IgG (10)].

After adding the FITC solution, the resulting solution was rotated in dark for 90 min at room temperature. Excess FITC was removed by dialysis against PBS four times. The ratio of the fluorescein dye to protein of the labeled antibody can be estimated by measuring the absorbance values at OD495 nm and OD280 nm.

### Modified-Sandwich ELISA for Detection of TFF3 Peptides

A sandwich ELISA was established based on our developed ELISA using the FITC and anti-FITC detection system [47]. The ELISA plate (Costar) was coated with purified anti-TFF3 mAbs (1 μg/ml) in carbonate/bicarbonate coating buffer of pH 9.6 at 4 °C overnight. After washing, the plate was blocked with 2% skimmed milk in PBS at 37 °C for 1 h. Various concentrations of the dimeric TFF3 or the saliva samples were then added and the plates were incubated for 1 h. FITC-labeled anti-TFF3 mAbs (1 μg/ml) were added and the plates were incubated at 37 °C for 1 h. HRP-conjugated sheep anti-FITC antibodies (Thermo Scientific) were used to detect the bound antibodies at 37 °C for 1 h. Thereafter, TMB substrate (Invitrogen) was added. The reaction was stopped using 1 N HCl and the absorbance was measured at OD450 nm.

## Abbreviations

ELISA: Enzyme-linked Lmmunosorbent Assay
FITC: Fluorescein Isothiocyanate
HRP: Horseradish Peroxidase
IgG: Immunoglobulin G
mAb: Monoclonal Antibody
OSCC: Oral Squamous Cell Carcinoma
SDS-PAGE: Sodium Dodecyl Sulfate Polyacrylamide Gel Electrophoresis
TFF: Trefoil Factor
TMB: 3,3′,5,5′-tetramethylbenzidine
